# Network Properties Influence Covariance Between Gene Expression and Fecundity Fitness of *Caenorhabditis elegans* in a Novel Laboratory Environment

**DOI:** 10.1093/gbe/evag180

**Published:** 2026-08-03

**Authors:** Tyler R Inskeep, Simon C Groen

**Affiliations:** Department of Botany and Plant Sciences, University of California, Riverside, USA; Department of Nematology, University of California, Riverside, USA; Institute for Integrative Genome Biology, University of California, Riverside, USA; Department of Botany and Plant Sciences, University of California, Riverside, USA; Department of Nematology, University of California, Riverside, USA; Institute for Integrative Genome Biology, University of California, Riverside, USA

**Keywords:** evolutionary systems biology, gene expression, fitness, microevolutionary dynamics, *Caenorhabditis elegans*

## Abstract

Gene expression bridges genotype and phenotype, shaping how organisms respond to their environments. Yet, it remains difficult to untangle how the architecture of gene regulatory networks (GRNs) influences the relationship between gene expression and fitness—especially in structured populations where genetic variants affecting both types of traits are inherited together. Here, we use publicly available data from wild *Caenorhabditis elegans* strains to link genome-wide gene expression levels to lifetime fecundity upon cultivation in a novel laboratory environment. Despite strong population structure caused by self-fertilization, selective sweeps, and hyperdivergent haplotypes, we find that a small subset of genes show significant covariance between their expression levels and fecundity fitness in laboratory cultivation conditions. These associations persist even after controlling for underlying genomic features. Genes that are older, show more tissue-specific expression patterns, and are particularly enriched in the germline and nervous system exhibit stronger covariance with fecundity in the new laboratory environment, consistent with known patterns of genetic divergence between wild and “domesticated” laboratory strains. Moreover, genes that are centrally positioned within GRNs, or are regulated downstream of certain transcription factors, show stronger associations with fitness. Together, these results suggest that genome structure and network topology jointly shape how variation in gene expression translates into fitness, shedding light on the early stages of adaptation to novel environments.

Significance Gene expression helps connect genetic differences to differences in survival and reproduction, but it remains unclear why changes in expression matter more for some genes than for others. By analyzing natural variation in gene expression and fecundity across wild strains of *Caenorhabditis elegans* upon cultivation in a laboratory environment, we found that expression-fitness covariance is generally weak but is stronger for genes that are tissue-specific, evolutionarily older, and more central in regulatory networks. These results show that the importance of gene expression for fitness depends not just on the gene itself, but on how that gene is embedded within broader biological systems.

## Introduction

Gene regulatory networks (GRNs) orchestrate organismal development and physiology, making them essential to understanding how phenotypes evolve. As populations diverge, changes in gene expression within GRNs can arise through both adaptive processes and neutral genetic drift; yet, the relative importance of each remains unresolved (True and Haag 2001; Hoekstra and Coyne 2007; Lynch 2007; Carroll 2008). Given that heritable variation in gene expression underlies many complex traits (Wang et al. 1999; Carroll 2000; Ayroles et al. 2009; Zhang et al. 2022), a key challenge is to determine how selective processes act on GRNs and shape phenotypic evolution (Romero et al. 2012).

A growing body of evidence suggests a combined role for network structure and gene history in the evolution of GRNs. Genes occupying central or highly connected positions within GRNs tend to show greater pleiotropy and are consequently subject to stronger constraints (Josephs et al. 2017). Younger genes, on the other hand, are often enriched at the periphery of GRNs, where constraints are relaxed, enabling expression divergence (Capra et al. 2010; Wei et al. 2016; Mähler et al. 2017; Defoort et al. 2018). Supporting these observations, the “omnigenic model” proposes that most gene expression differences in populations are influenced by trans-regulatory variants scattered throughout the genome, reinforcing the notion that GRNs function as distributed systems with many indirect influences (Liu et al. 2019). Despite these presumed constraints, regulatory divergence can still accumulate. For example, well-conserved orthologs, such as those conserved among species in the nematode genus *Caenorhabditis*, exhibit weak coupling between rates of sequence and expression evolution (Castillo-Davis et al. 2004), reinforcing the notion that compensatory drift and network reorganization might contribute substantially to GRN evolution (True and Haag 2001).

In multicellular organisms, functional interdependence between tissues constrains trait evolution because effects of mutations can propagate through shared signaling or regulatory networks that operate across tissues (Liang et al. 2018). Tissue-specific GRNs are believed to enhance functional flexibility by reducing pleiotropic consequences across multiple tissues. Supporting this hypothesis, tissue-specific genes are reported to diverge more rapidly at the sequence level, while their expression divergence is often limited, reflecting a potential constraint imposed by regulatory specificity (Zhang and Li 2004; Liao and Zhang 2006). Reproductive tissues stand out as hotspots for gene expression evolution, likely driven by sexual selection (Brawand et al. 2011; Fukushima and Pollock 2020; El Taher et al. 2021; Schuster et al. 2024). However, it remains unclear how tissue specificity interacts with regulatory architecture to shape the capacity of gene expression traits to evolve.

Comparative studies of gene expression divergence have advanced our understanding of GRN evolution, although these are often limited in their ability to disentangle adaptive changes from lineage-specific drift (reviewed by (Price et al. 2022)). Population-level studies offer a complementary approach by shedding light on how gene expression levels covary as quantitative traits with fitness component metrics (Groen et al. 2020; Koch and Guillaume 2020; Ahmad et al. 2021; Gupta et al. 2025; Henry and Stinchcombe 2025). Yet, results vary across systems. For example, population-scale variation in transcript abundance displayed negative covariance with fitness for most genes in rice cultivated under traditional paddy field conditions (Groen et al. 2020; Gupta et al. 2025), whereas opposite trends were observed in field-sampled salmonid fish (Ahmad et al. 2021). These mixed patterns challenge the expectation from comparative studies that stabilizing selection should constrain gene expression divergence (Bedford and Hartl 2009; Kalinka et al. 2010; Chen et al. 2019; El Taher et al. 2021), raising critical questions about how GRNs diverge across scales and species. To resolve these questions, patterns of covariance between genes’ expression levels and fitness must be interpreted in the context of the GRNs in which genes are embedded. This systems-level perspective offers a promising bridge between population-scale associations and macroevolutionary patterns observed across species. In doing so, it allows us to ask whether the same principles that govern GRN divergence over long timescales also manifest in microevolutionary patterns of gene expression-fitness covariance within a single generation.

In this study, we reanalyze publicly available genome-wide gene expression and phenotypic data (Hahnel et al. 2018; Zhang et al. 2021, 2022) to investigate patterns of transcript level-fitness covariance in a population of field-collected strains of the nematode *Caenorhabditis elegans*. These wild isolates had been maintained in the laboratory for only a limited number of generations, making them a valuable system for exploring how gene expression variation maps onto fitness in a novel environment. The laboratory environment exhibits several key differences from the wild: it is homogeneous, nutrient-rich, free of natural enemies, and characterized by constant temperature and light cycles (Félix and Braendle 2010; Sterken et al. 2015). These artificial environmental conditions impose strong selective pressure on traits related to sensory processing, feeding behavior, and developmental and reproductive timing (Weber et al. 2010; Sterken et al. 2015). As a result, the phenotypic differences observed between laboratory-adapted and wild strains provide a comparative framework with which to interpret gene expression-fitness covariance as organisms respond to these simplistic yet novel environmental conditions.

However, a key challenge in interpreting trait-fitness associations in *C*. *elegans* is its strong population structure driven by hermaphroditic reproduction. Predominant selfing reduces effective recombination and gene flow, leading to strong linkage disequilibrium and creating large linked haploblocks that are inherited together (Rockman and Kruglyak 2009; Andersen et al. 2012). Consequently, genetic diversity in *C*. *elegans* is extremely low on a global scale (Crombie et al. 2019) and is instead concentrated in hyperdivergent regions (HDRs) characterized by extensive structural variation and maintained by balancing selection (Lee et al. 2021). Reduced recombination in HDRs makes it difficult to disentangle causal loci from linked background variation in mapping studies, especially considering that many trait associations map to variants within these regions (Zhang et al. 2022). Additionally, the prevalence of linked selection—including both background selection and chromosome-scale selective sweeps—can skew patterns of allele frequency change and obscure true genotype–phenotype relationships (Rockman et al. 2010; Andersen et al. 2012; Cutter et al. 2019; Zhang et al. 2021). Together, these features make it critical to consider the impact of population structure and genomic context when interpreting gene expression-fitness covariance in *C*. *elegans*.

In keeping with the omnigenic model of gene expression (Liu et al. 2019), transcript abundance is a polygenic trait and thus we expect that most transcripts should exhibit little or insignificant covariance with fitness (Kingsolver et al. 2001). Thus, rather than focusing on individual transcripts whose abundance covaries with fitness, we ask whether specific features of GRNs systematically influence the strength of gene expression-fitness covariance. In particular, we explore whether the expression of older genes, genes with more central positions in GRNs, or tissue-specific genes is more strongly aligned with fecundity, and whether these patterns support reproductive tradeoffs shaped by the life history of *C*. *elegans* (Cutter 2004; Murray and Cutter 2011). By identifying these systems-level patterns, our study provides new insight into the constraints and flexibilities imposed by regulatory network architecture on the microevolutionary potential of population-wide variation in gene expression.

## Results

### Weak and Variable Covariance Between Genome-wide Gene Expression Patterns and Fecundity Fitness in *C*. *elegans* in Laboratory Conditions

To investigate how transcript levels covary with fitness in a population of genetically diverse, wild-caught *C*. *elegans* upon cultivation in a laboratory environment, we leveraged publicly available transcriptome data from life stage-synchronized worms of 207 strains curated by the *Caenorhabditis* Natural Diversity Resource (CaeNDR; Crombie et al. 2024). These strains represent species-wide genetic diversity and have been characterized by genome resequencing (Lee et al. 2021), genome-wide gene expression profiling (Zhang et al. 2022), and measurements of total lifetime fecundity (TLF) as a key fitness component (Hahnel et al. 2018; Zhang et al. 2021). Wild worms were reared in a novel common laboratory environment on solid agar plates and collected for measuring transcript abundance during the onset of adulthood, around the first embryo-laying event (Zhang et al. 2022), a critical time point for assessing reproductive fitness. TLF was calculated by summing progeny produced over four days by worms reared in two standard laboratory environments: one, in liquid culture, fed with *Escherichia coli* strain HB101 (Hahnel et al. 2018); and the second, on solid agar plates, fed with *E*. *coli* strain OP50 (Zhang et al. 2021). These publicly available datasets provided us with an opportunity to infer direct links between gene expression patterns and fitness outcomes.

We applied genotypic selection analyses (Rausher 1992) to estimate selection differentials for 25,849 transcripts, representing approximately 16,094 genes. Two metrics were calculated: *S*, representing directional selection (whether transcript abundance covaries positively or negatively with fitness), and *C*, representing stabilizing or diversifying selection (selection for or against population-level variance in gene expression, respectively). We initially calculated *S* and *C* across a panel of 66 strains for which fecundity was estimated in both culture conditions, to understand the environmental dependency of this fitness component and transcript level-fitness associations. Although fecundity fitness was only moderately correlated (Spearman's *ρ* = 0.352, *P* = 0.003715; Fig. S1a), directional selection coefficients (*S*) were more strongly correlated across environments (Spearman's *ρ* = 0.632, *P* < 2.2 × 10^−16^; Fig. S1b), suggesting that transcriptome-wide fitness associations might capture shared components of reproductive performance across laboratory culturing conditions. Given this concordance, we decided to focus subsequent analyses on *S* estimated using fecundity from the liquid culture dataset, as its larger sample size increased the statistical power of our analyses: 193 strains assayed in liquid had published transcriptome data, compared to 78 strains on agar.

**Fig. 1.**
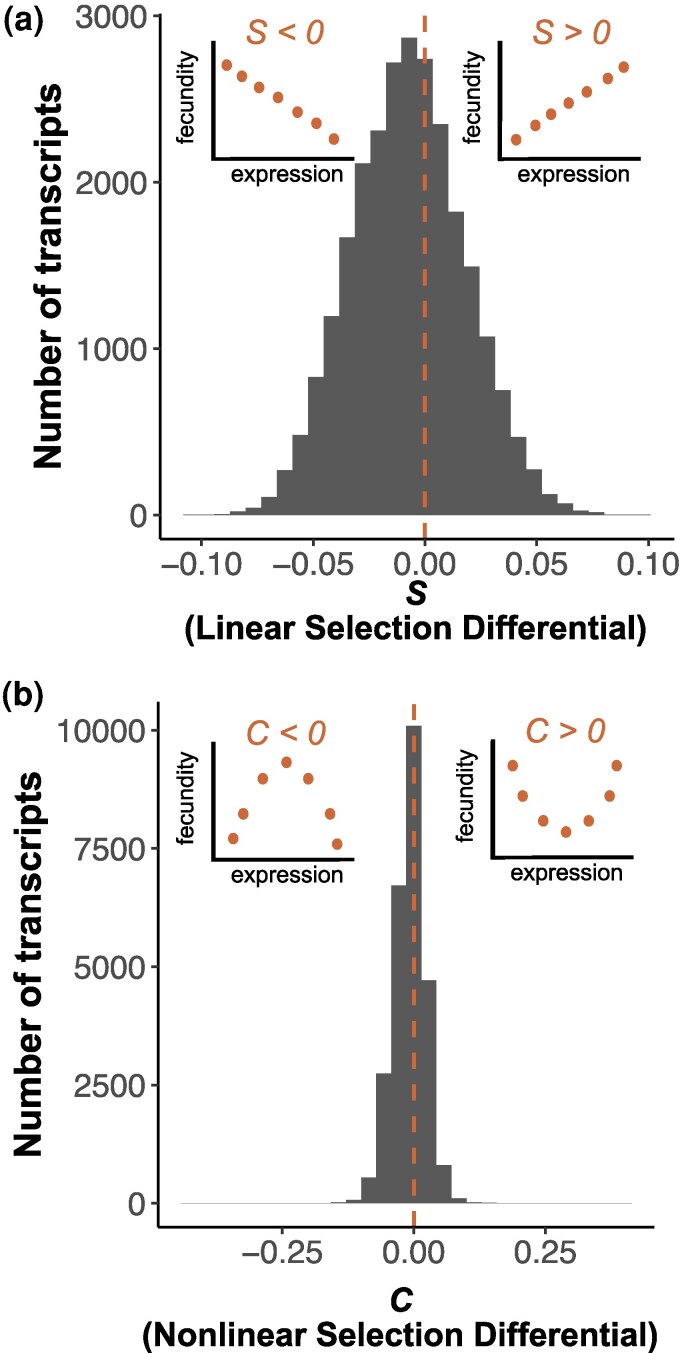
The strength and pattern of transcript level-fecundity fitness covariance of *C*. *elegans* in a novel laboratory environment. a) Distribution of linear selection differentials (*S*) on genome-wide gene expression. Vertical dotted orange line represents *S* = 0. b) Distribution of quadratic/nonlinear selection differentials (*C*) on genome-wide gene expression. Vertical orange line represents *C* = 0.

Across all transcripts, patterns of gene expression-fitness covariance in the larger panel were generally weak, with *|S|*_median_ = 0.018. Estimates of *S* were more often negative than positive (Mann–Whitney *U*-test, two-sided, *P* < 2.2 × 10^−16^; Fig. 1a), suggesting that the expression of most genes is inversely correlated with fitness in the laboratory. Additionally, we observed a bias toward negative estimates of *C* (Mann–Whitney *U*-test, two-sided, *P* < 2.2 × 1^−16^; Fig. 1b). We observed a similar bias with *C* estimated from fitness on agar (Mann–Whitney *U*-test, two-sided, *P* < 2.2 × 1^−16^), suggesting that in the new laboratory environment, expression variance tends to be disadvantageous, consistent with prior findings that suggest pervasive weak stabilizing selection influences the evolution of genome-wide gene expression patterns in *C*. *elegans* (Denver et al. 2005; Zhang et al. 2022; Bell et al. 2025). However, estimates of *C* for transcript levels varied across environments (Spearman's *ρ* = 0.299, *P* < 2.2 × 10^−16^; Fig. S1c). For this reason, and given the complex nature of interpreting selection on trait variance (Mitchell-Olds and Shaw 1987), we focus primarily on interpreting linear transcript level-fitness associations approximated by *S*. Full selection differentials for each transcript across both environments are reported in Table S1.

Seven transcripts retained significant *S-*values after Bonferroni correction (*P* < 3.87 × 10^−7^; Fig. S1). Among these, we observed a positive covariance between fecundity and the expression of *ugt-9*, which encodes a UDP-glycosyltransferase known to be involved in detoxifying benzimidazole-like compounds (Sharma et al. 2024). Conversely, we observed a negative covariance between fecundity and the expression of *B0348*.*2*, encoding a predicted zinc ion-binding protein; *ttn-1* (*Titin-1*), which encodes a key structural component of muscle filaments (Forbes et al. 2010); *feh-1*, encoding an amyloid binding protein known to regulate pharyngeal pumping (Zambrano et al. 2002); *clec-37*, encoding a C-type lectin; *F25A2*.*1*, a gene with predicted contributions to lipid metabolism; and *nhr-114*, which encodes a nuclear hormone receptor responsive to nutritional cues (Gracida and Eckmann 2013; Giese et al. 2020; Qin et al. 2022). We will discuss potential relationships between variation in expression for several of these transcripts and fecundity later. It was further notable that no transcripts retained significant *C* values after applying Bonferroni correction. Taken together, these results highlight that, while levels of most transcripts display only weak covariance with fecundity fitness, expression of a few individual genes may make outsized contributions to fitness.

To validate our estimates of transcript level-fitness covariance, we integrated our measurements of *S* and *C* with previously identified QTL associated with TLF (Zhang et al. 2022). Of the 17 QTL, ten encoded at least one transcript with a significant value for *S*, and two encoded a transcript significant for *C*, prior to Bonferroni correction (Fig. S3; Table S5). This general pattern was also observed when considering models based on fecundity fitness from agar. Notably, *nhr-114* is located inside a fecundity-associated QTL and one of its transcripts retained significance after Bonferroni correction (Figs. S2g and S3a). This integration reinforces the biological relevance of our genotypic selection models and highlights potential regulatory mechanisms underlying fitness variation.

Univariate genotypic selection differentials (*S* and *C*) reflect both direct contributions of trait variation to fitness as well as indirect contributions due to genetic correlations between traits (Lande and Arnold 1983). To examine how coordinated patterns of gene expression covary with fecundity in the novel laboratory environment, we conducted principal component (PC) analysis on genome-wide gene expression. The top 22 PCs, individually explaining at least 0.5%, and collectively explaining 59.02%, of variation in transcript abundance, were subjected to multivariate regression so that direct linear (*β*) and quadratic (*Ɣ*) selection gradients could be estimated (Fig. S4a–d; Table S2). Among these PCs, eight exhibited significant linear covariance with fecundity (*β*, *P* < 0.05; Fig. S4c), and one showed significant quadratic covariance with fecundity (*Ɣ*_PC1_ = 0.136, *P* = 0.0208; Fig. S4d). However, after applying Bonferroni correction to our multivariate models (*P* < 0.0022), only two PCs maintained significant linear selection gradients (*β*_PC4_ = 0.0858, *P* = 9.33 × 10^−8^; *β*_PC11_ = −0.04907, *P* = 0.00169), while none retained significance for *Ɣ*. These results are concordant with the patterns of pervasive weak selection that emanated from our univariate selection analyses (Fig. 1) and suggest that selection may target coordinated expression patterns of multiple genes rather than abundance of individual transcripts, consistent with the omnigenic model of expression evolution (Liu et al. 2019).

### Estimates of Gene Expression-fitness Covariance are not Biased Toward Major Axes of Population and Genomic Variation

To confirm that our selection differential estimate *S* reflects biological patterns rather than artifacts of population structure, we first repeated the genotypic selection analysis after removing 14 Hawaiian strains, which are genetically divergent from the rest of the population (Lee et al. 2021) and exhibit lower relative fecundity in laboratory environments (Zhang et al. 2021). These strains could potentially confound trait-fitness covariance due to their outlier status. However, their exclusion did not meaningfully alter selection differential estimates, as shown by strong concordance between full and filtered models (Spearman *ρ* = 0.9668, *P* < 2.2 × 10^−16^) (Fig. S5a; Table S4). As a second test of population structure, we repeated the analysis only using 99 European strains and again observed high concordance (Spearman *ρ* = 0.8075, *P* < 2.2 × 10^−16^) (Fig. S5b; Table S4). Together, these analyses indicate that population structure in the CaeNDR panel does not substantially bias genome-wide estimates of transcript level-fitness covariance.

We next asked whether broad genomic context influences the strength of transcript level-fitness covariance. Distinguishing features of the *C*. *elegans* genome, such as chromosome-scale selective sweeps and HDRs, are enriched in chromosome centers, which exhibit reduced recombination (Andersen et al. 2012; Lee et al. 2021). These features are also associated with gene expression variation and fecundity (Zhang et al. 2021, 2022). To test whether high estimates of transcript level-fitness covariance were concentrated in particular genomic contexts, we modeled the magnitude of transcript level-fitness covariance (*|S|*) as a function of chromosome identity, chromosomal domain, and their interaction. This analysis revealed significant variation in *|S|* across chromosomes (*F* = 31.94, *P* < 2.2 × 10^−16^), and a weaker but significant effect of chromosomal domain, with *|S|* marginally higher in chromosome centers (*F* = 8.12, *P* = 0.004). However, the interaction term was not significant (*F* = 0.8132, *P* = 0.54; Fig. S5c). Thus, broad genomic position has a detectable but modest association with *|S|*, motivating more direct tests of whether selective sweep history or HDR frequency explain genome-wide patterns in transcript level-fitness covariance.

First, we tested whether selective sweep history may explain the observed genome-wide patterns. Each gene was assigned a sweep burden, defined as the number of strains in which that gene overlapped a swept haplotype based on strain-wise selective sweep annotations (Lee et al. 2021). Genes were grouped into five equal-sized bins by sweep burden. If shared sweep history were driving transcript level-fitness covariance estimates, genes overlapping swept haplotypes in more strains would be expected to show systematically altered *|S|* for their expression. However, *|S|* did not vary across genes with variable sweep burden (Kruskal–Wallis test, *P* = 0.0861; Fig. S5d), suggesting that selective sweeps do not explain the genome-wide landscape of transcript level-fitness covariance.

Second, we tested whether HDRs could bias transcript level-fitness covariance estimates. Genes located within strain-specific HDRs can have unreliable expression estimates because high sequence divergence can reduce read-mapping accuracy; accordingly, transcript abundance estimates for genes within HDRs must be excluded from affected strains (Lee et al. 2021; Zhang et al. 2022). This creates a potential source of bias because estimates of *S* for expression of genes overlapping HDRs are estimated from smaller subsets of the population. To address this concern, we identified segregating HDRs across the 172-strain panel and assigned each gene an HDR burden, defined as the number of strains in which that gene overlapped an HDR. *|S|* did not differ significantly among HDR burden categories (Kruskal–Wallis test, *P* = 0.341; Fig. S5e), suggesting that HDRs, and the uncertainty of gene expression estimates that accompany them, do not explain genome-wide patterns of transcript level-fitness covariance either.

Consistent with prior findings in *C*. *elegans* (Bell et al. 2023, 2025), these results suggest that although genomic features like HDRs may influence individual transcript level-fitness associations, they do not drive genome-wide patterns, further supporting the robustness of our global estimates. We therefore proceed with analyses of global patterns in *S*, while controlling for genomic context in targeted downstream analyses where relevant.

### Network Architecture Constrains Covariance Between Gene Expression and Fecundity in Laboratory Conditions

The abundance of each transcript reflects regulatory inputs from chromatin state, transcription factor (TF) binding, and broader network architecture. Having found that our selection differential estimates (*S*) were not primarily driven by population structure or broad genomic features, we next asked instead whether these estimates were influenced by regulatory architecture. If our selection models captured biologically meaningful variation, then genes located in key regions of the network may show stronger associations between expression and fecundity in laboratory conditions. To test this, we integrated estimates of transcript level-fitness covariance (*S*) with chromatin accessibility data from L4-stage worms (Serizay et al. 2020) and TF-target relationships inferred from time-course transcriptomes in adult worms (Suriyalaksh et al. 2022).

**Fig. 2.**
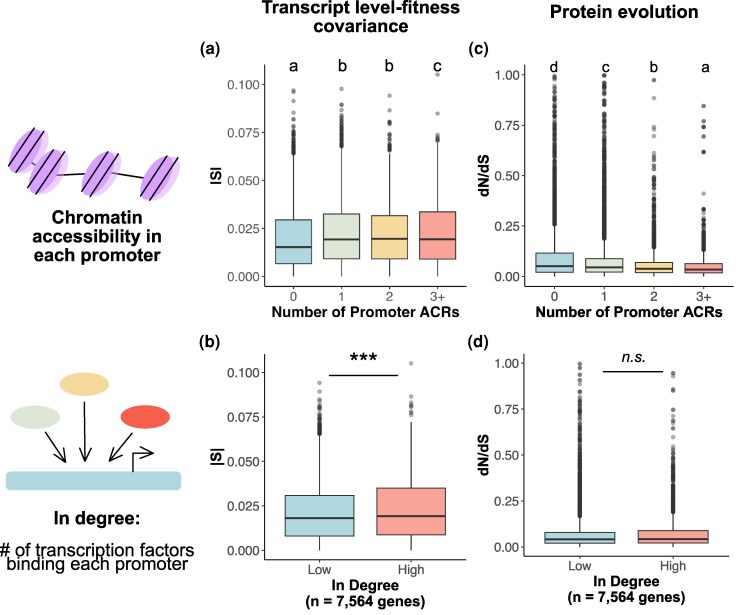
Network architecture shapes patterns of gene expression-fitness covariance. a and c) Genes with more accessible chromatin regions (ACRs) show (a) stronger expression-fitness covariance (*χ*^2^ = 151.74, *P* < 2.2 × 10^−16^), while (c) their protein-coding sequences show reduced evolutionary rates (*χ*^2^ = 363.8, *P* < 2.2 × 10^−16^). b and d) Genes regulated by more transcription factors display (b) stronger expression-fitness covariance (*P* = 0.01366), while (d) their protein-coding sequences do not differ in evolutionary rates *(P* = 0.0802). Boxplots show the median and interquartile range. For comparisons with more than two groups, overall differences were tested using Kruskal–Wallis tests, followed by Dunn's post hoc tests with Bonferroni correction. Letters indicate post hoc significance groups; groups that do not share a letter differ at adjusted *P* < 0.05, with “*a*” assigned to the group with the lowest median. For two-group comparisons, significance was assessed using Mann–Whitney *U*-tests.

We first tested whether local regulatory complexity, measured by the number of accessible chromatin regions (ACRs) in each gene's promoter, was associated with the strength of transcript level-fitness covariance (*|S|*). Expression of genes with increasingly accessible promoter chromatin showed stronger *|S|* (Fig. 2a; Kruskal–Wallis *χ*^2^ = 151.74, *P* < 2.2 × 10^−16^) and a similar pattern was observed for enhancer-associated chromatin regions (Fig. S6b; Kruskal–Wallis *χ*^2^ = 132.83, *P* < 2.2 × 10^−16^). Thus, genes with more broadly accessible regulatory landscapes show stronger covariance between transcript abundance and fecundity of worms in the novel laboratory environment.

This relationship extended from local chromatin architecture to broader network position. Expression of genes with high regulatory in-degree, defined as genes predicted to be regulated by more TFs, showed stronger *|S|* than that of genes with low in-degree (Fig. 2b; Mann–Whitney *U*-test, *z* = −3.687, *P* = 2.268 × 10^−4^). By contrast, TF out-degree, defined as the number of target genes regulated by each TF, was not associated with variance in *|S|* among TFs (Fig. S6f; Kruskal–Wallis *χ*^2^ = 1.953, *P* = 0.3765). Together, these patterns are consistent with a polygenic architecture of fecundity (Zhang et al. 2021), in which gene expression-fitness covariance is distributed across many highly regulated target genes, rather than concentrated among a few highly connected TFs (Liu et al. 2019).

Interestingly, these patterns related to network architecture seemed to distinguish |*S*| as an estimate of microevolution from signatures of longer-term sequence evolution. To test whether genes with stronger expression-fitness covariance were also unique at the sequence level, we compared regulatory architecture with two complementary evolutionary metrics: *dN/dS*, which reflects protein-coding evolutionary constraint, and Tajima's *D*, which captures departures from neutral patterns of within-species nucleotide variation. Genes marked by increasingly accessible chromatin exhibited lower *dN/dS* values (Fig. 2c;  Fig. S6c, Kruskal–Wallis *P* < 2.2 × 10^−16^). Thus, genes with more broadly accessible regulatory landscapes not only showed stronger expression-fitness covariance but also tended to encode more slowly evolving proteins.

This relationship did not extend uniformly to network position. High in-degree genes, despite showing stronger *|S|* for their expression, did not differ significantly in *dN/dS* (Mann–Whitney *U*-test, *P* = 0.08082; Fig. 2d). Similarly, TF out-degree was not associated with *dN/dS* (Kruskal–Wallis *χ*^2^ = 4.8583, *P* = 0.4346; Fig. S6g). Additionally, no differences in Tajima's *D* were observed across each network architecture trait (Fig. S6a and d, e, and h), implying that these patterns are not simply explained by broad deviations in within-species nucleotide diversity. Together, these results suggest that network connectivity and chromatin accessibility capture unique facets of network architecture, both of which are associated with heightened covariance between gene expression and fecundity in laboratory conditions.

### Tissue-specific Heterogeneity of Gene Expression-fitness Covariance

Regulatory architecture can reveal principles of how gene expression is calibrated, but genes ultimately function in cellular and tissue-specific contexts. Given that fecundity is a complex organismal trait that is influenced by coordinated physiological processes across multiple organs, we set out to investigate how tissue specificity influences the potential for genes’ expression levels to evolve in a novel environment. Toward this goal, we calculated *τ* (tau) for each gene in the *C*. *elegans* genome. *τ* ranges from 0 (broad/constitutive expression) to 1 (tissue-specific expression), providing a quantitative measure of expression breadth (Yanai et al. 2005). Using tissue-specific transcriptome data collected for four major tissues in L4-stage worms (Serizay et al. 2020), we found that expression of most genes in *C*. *elegans* is enriched in a limited number of tissues (Fig. S7a; *τ* estimates can be found in Table S6).

**Fig. 3.**
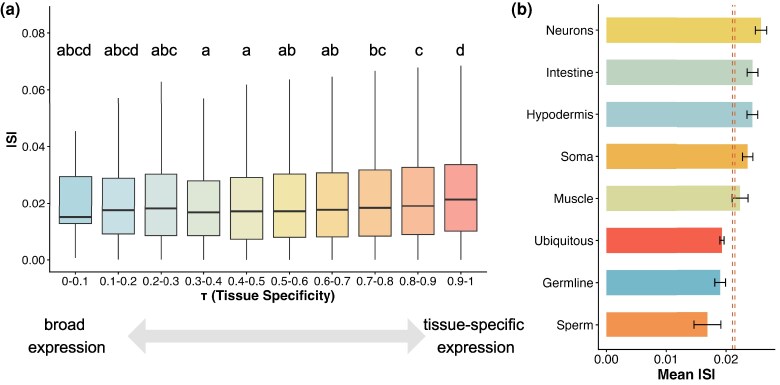
Expression of tissue-specific transcripts covaries more intensely with fecundity in the laboratory. a) Expression of genes with greater degrees of tissue specificity (higher *τ*) shows stronger fitness covariance (*|S|*; Kruskal–Wallis, *χ*^2^ = 119.16, *P* < 2.2 × 10^−16^). Boxplots show the median and interquartile range. Global differences were tested using a Kruskal–Wallis test, followed by Dunn's post hoc tests with Bonferroni correction. Letters indicate post hoc significance groups; groups that do not share a letter differ at adjusted *P* < 0.05, with “*a*” assigned to the group with the lowest median. b) Expression of germline- and sperm-specific genes displays weaker fitness covariance, while expression of neuronal, intestinal, and hypodermal genes shows stronger fitness covariance. Bars represent mean |*S*| values and error bars represent the 95% confidence intervals (CIs). Vertical dashed orange lines represent the 95% CIs for |*S*| across all genes in the genome.

Genes with higher tissue specificity showed stronger covariance between expression and fecundity fitness (*S*: Kruskal–Wallis *χ*^2^ = 119.16, *P* < 2.2 × 10^−16^, Fig. 3a). Tissue-specific genes did not vary substantially in Tajima's *D* values (Kruskal–Wallis *χ*^2^ = 20.833, *P* = 0.0132, Fig. S7b), but did exhibit elevated *dN/dS* levels (Kruskal–Wallis *χ*^2^ = 248.74, *P* < 2.2 × 10^−16^; Fig. S7c), consistent with accelerated protein evolution. These patterns align with prior studies showing that tissue-specific genes evolve faster at the protein level (Zhang and Li 2004). They also suggest that tissue-specific genes may represent a class of loci where both protein sequence evolution and expression variation are closely linked to organismal performance. Stronger fitness covariance for expression of tissue-specific and patterned genes further echoes results of comparative embryonic studies in *Caenorhabditis*, where tissue-specific genes showed reduced expression conservation across species (Large et al. 2025).

**Fig. 4.**
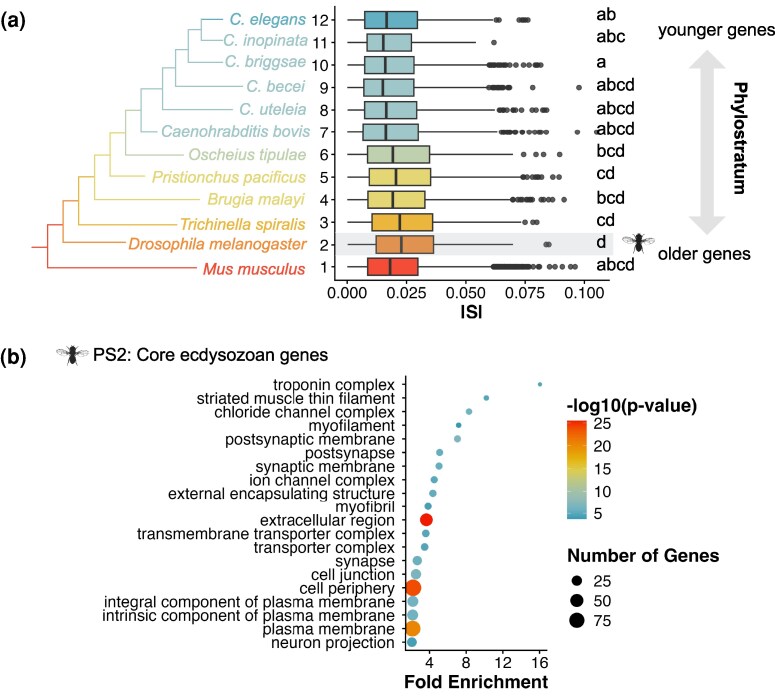
The landscape of transcript expression-fitness covariance is shaped by gene age. a) Genes were assigned relative ages, i.e. they belonged to different phylostrata (PS1-12 along the y axis), based on their presence in orthogroups that were conserved in the phylogeny estimated by Ma and Zheng (2023). Expression of older genes shows stronger fitness covariance (*|S|*; Kruskal–Wallis *χ*^2^ = 191.97, *P* < 2.2 × 10^−16^). Boxplots show the median and interquartile range. Global differences were tested using a Kruskal–Wallis test, followed by Dunn's post hoc tests with Bonferroni correction. Letters indicate post hoc significance groups; groups that do not share a letter differ at adjusted *P* < 0.05, with “*a*” assigned to the group with the lowest median. b) PS2, corresponding to gene families generally conserved across ecdysozoans, showed the strongest transcript level-fitness covariance and was enriched for genes localizing to the neuromuscular junction.

The *τ* analysis did not reveal, however, whether this pattern was driven equally by all tissues. To ask whether tissue-enriched transcriptomes differed in their average association with fecundity, we retrieved tissue-specific gene sets from (Serizay et al. 2020) and calculated 95% confidence intervals for *|S|* for each tissue's transcriptome (Fig. 4c). Our analysis revealed that transcript abundances for neuronal, intestinal, hypodermal, and somatic tissue-specific genes covaried more strongly with fecundity, suggesting that expression variation in these physiological systems contributes disproportionately to fecundity differences among strains in laboratory conditions. Transcript abundances for muscle-specific genes showed values of *|S|* comparable to the genome-wide average. In contrast, transcript abundances for germline- and sperm-specific genes as well as those for ubiquitously expressed genes experienced weaker covariance with fecundity, suggesting that expression differences among strains in these gene sets explain less of the observed fecundity variation under these conditions.

### Older Genes Show Stronger Transcript Level-fitness Covariance and Broader Regulatory Integration

Having found that transcript level-fitness covariance is associated with regulatory architecture and tissue specificity, we became curious whether these patterns might vary with gene age. Gene age provides a useful test case because it links two expectations that are not obviously aligned. Older genes are expected to be more deeply integrated into GRNs, whereas younger genes are thought to be more tissue-specific and peripheral to GRNs. Because our analyses found stronger transcript level-fitness covariance for both tissue-specific and highly regulated genes, we asked whether gene age distinguishes these different routes to fecundity-associated expression variation.

To test this, we used phylostratigraphy, a comparative genomic approach that estimates gene age based on the phylogenetic distribution of homologous gene families. Genes assigned to lower phylostrata have homologs distributed across deeper evolutionary lineages, whereas genes assigned to higher phylostrata are more taxonomically restricted and are inferred to have emerged more recently (Domazet-Loso et al. 2007). Using phylostratum (PS) annotations for *C*. *elegans* from (Ma and Zheng 2023), we compared transcript level-fitness covariance across gene age classes.

Fitness covariance (as estimated by *|S|*) was significantly stronger for expression of older genes (Kruskal–Wallis *χ*^2^ = 191.97, *P* < 2.2 × 10^−16^; Fig. 4a). The strongest covariance was observed for expression of genes in PS2, corresponding to core ecdysozoan genes, while the weakest covariance occurred for expression of PS10 genes, shared between *C*. *elegans* and its close relative *C*. *briggsae*. PS2 genes were enriched for Gene Ontology (GO) cellular compartments essential to neuromuscular function, such as the troponin complex, synaptic membrane, and neuron projections (Fig. 4b). These findings align with previous reports that younger genes are less likely to develop essentiality and contribute to fitness in *C*. *elegans* (Ma et al. 2024). They also support previous observations that laboratory environments impose strong selective pressure on behavior, feeding, and sensory processing during adaptation (Sterken et al. 2015).

We next asked whether younger genes showed the expected signatures of restricted regulatory integration. Consistent with previous models of young gene evolution, younger genes exhibited, on average, higher tissue specificity (*τ*; Kruskal–Wallis *χ*^2^ = 1352.8, *P* < 2.2 × 10^−16^; Fig. S8a), and were associated with fewer ACRs in their promoters (Kruskal–Wallis *χ*^2^ = 650.69, *P* < 2.2 × 10^−16^′ Fig. S8b). These results suggest that gene age separates two related, but distinct routes to fecundity-associated expression variation. Tissue specificity is associated with stronger transcript level-fitness covariance overall, but younger tissue-specific genes appear to occupy more restricted regulatory contexts. By contrast, older genes show stronger covariance with fecundity, consistent with their deeper integration into conserved physiological and regulatory networks.

### Functional Enrichment of Univariate and Multivariate Selection Models

Having identified transcript classes with stronger expression-fitness covariance, we then wished to find out which biological processes were associated with the direction of covariance. Whereas *|S|* captures the strength of transcript level-fitness covariance, signed *S* distinguishes transcripts whose abundance covaries positively or negatively with fecundity. This distinction is important because positive and negative covariance may reflect different physiological strategies relevant for fitness in the laboratory environment.

**Fig. 5.**
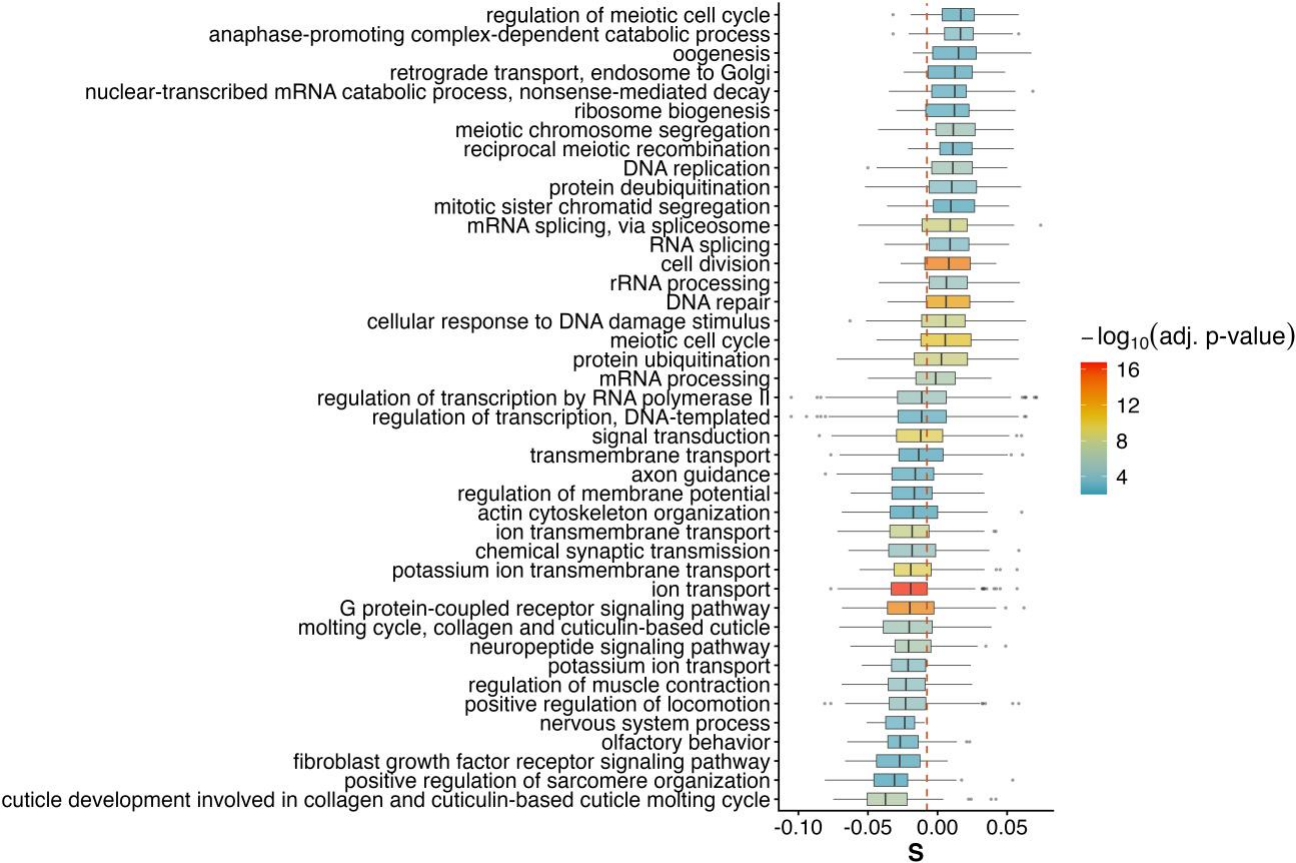
Functional enrichment of signed transcript level-fitness covariance highlights reproductive and neuromuscular tradeoffs. Gene Ontology (GO) biological processes with larger-than-average median *S-*values for the expression of underlying genes (Mann–Whitney *U*-test, two-sided, *P* < 1.15 × 10^−6^). Vertical dotted orange line represents the overall median selection differential *S*_median_ = 0.018. Boxplots represent the median and interquartile ranges.

Following Bonferroni correction, we identified 45 GO biological processes whose constituent genes showed stronger-than-average signed covariance with fecundity (*S;* Mann–Whitney *U-*tests, Bonferroni-adjusted *P* < 1.15 × 10^−6^; Fig. 5; Table S7). Among these, expression of genes involved in reproductive processes (e.g. regulation of meiotic cell cycle, reciprocal meiotic recombination, meiotic chromosome segregation) tended to covary positively with fecundity. By contrast, expression of genes involved in behavior and neuromuscular function (e.g. chemical synaptic transmission, axon guidance, neuropeptide signaling, regulation of muscle contraction) tended to covary negatively with fecundity.

These opposing functional patterns suggest that higher fecundity in the laboratory environment is associated with increased expression of reproductive and cell-cycle-associated genes and reduced expression of behavioral, sensory, neuromuscular, and developmental programs. This aligns with prior work showing that that laboratory-adapted *C*. *elegans* strains can gain fitness through accelerated reproductive output and reduced investment in behaviors that are costly under standard culture conditions, including aggregation, aerotaxis, social feeding, and sensory responses (de Bono and Bargmann 1998; Rogers et al. 2003; Gray et al. 2004; Chang et al. 2006; Macosko et al. 2009; McGrath et al. 2009; Félix and Braendle 2010; Bendesky et al. 2011; Andersen et al. 2014). They further suggest that the “laboratory domestication” of *C*. *elegans* might involve broad transcriptional reprogramming (Rockman et al. 2010; Andersen et al. 2014; Sterken et al. 2015), mirroring patterns observed in domesticated crops such as maize (*Zea mays*; (Swanson-Wagner et al. 2012) and tomato (*Solanum lycopersicum*; (Koenig et al. 2013).

We further wanted to know whether these functional patterns were also apparent in coordinated axes of transcriptome variation, returning to our PC-based multivariate analysis. GO enrichment of the top 200 genes loading into significant PCs highlighted multiple faces of RNA metabolism (Fig. S4e–k), some of which also appeared in the univariate analysis (Fig. 5). Thus, the functional signals recovered from univariate analyses are complemented by multivariate evidence that fecundity-associated expression variation is linked to coordinated biological processes.

### Gene Expression-fitness Covariance Highlights Tradeoffs Between Reproductive Strategies

**Fig. 6.**
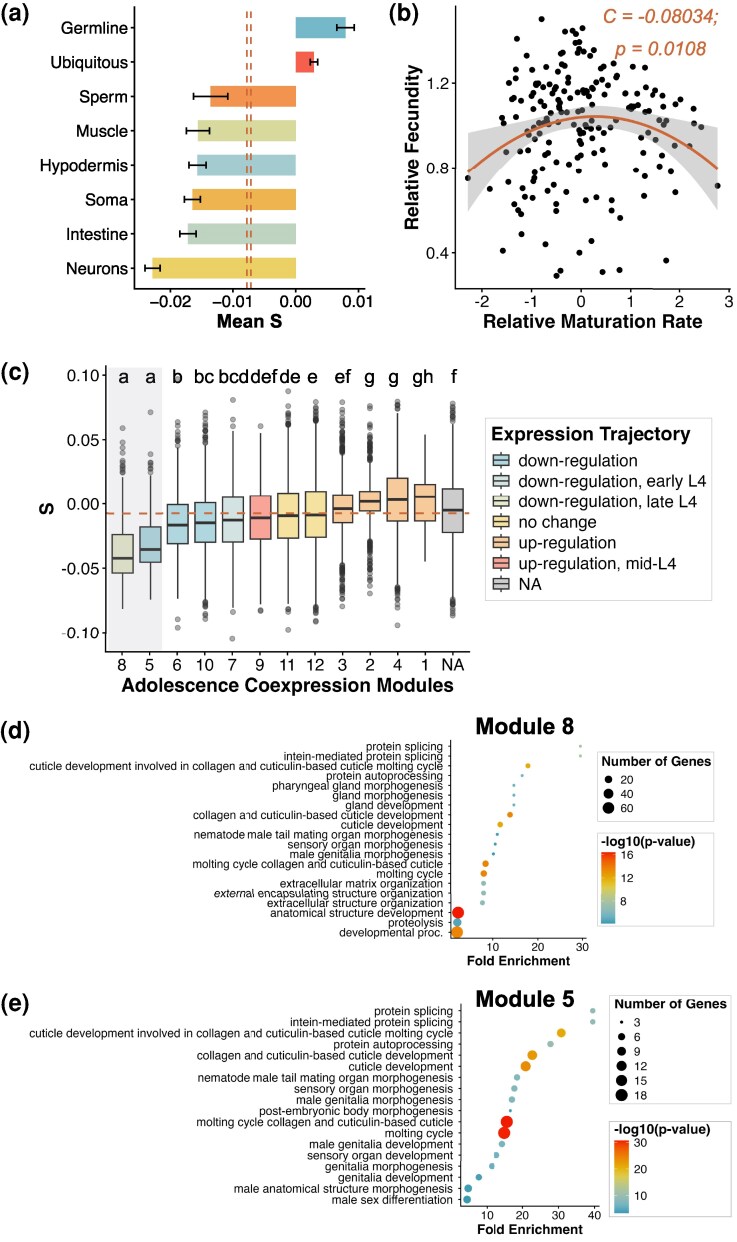
Patterns of transcript expression-fitness covariance highlight tradeoffs between nematode reproductive strategies. a) Transcript expression-fitness covariance for germline-specific and ubiquitous genes is positive on average, while this is negative on average for all other tissue-specific gene sets. Bars represent mean *S*-values with 95% confidence intervals (CIs). Vertical dashed orange lines represent 95% CIs for *S* across all genes in the genome. b) Covariation between time to maturity and fecundity suggests weak stabilizing selection on developmental timing in *C*. *elegans* (*C* = −0.0803 ± 0.0312, *P* = 0.0108). c) Average transcript expression-fitness covariance across coregulated clusters of genes with dynamic expression patterns during the adolescence transition as determined by Snoek et al. (2014). While most clusters have mean *S*-values near the genome-wide median selection differential (horizontal dashed orange line; *S* = −0.018), expression of genes in clusters 5 and 8 shows more strongly negative fitness covariance (Kruskal–Wallis test with Dunn's post hoc tests, *P* < 2.746 × 10^−4^). Boxplots show the median and interquartile range. d and e) Results of Gene Ontology (GO) biological process enrichment analyses for genes in (d) cluster 8 and (e) cluster 5 suggest that reduced expression of genes that promote development and molting is associated with elevated fecundity.

The signed direction of transcript level-fitness covariance allowed us to ask whether tissue-specific transcriptomes were associated with distinct reproductive strategies. After unfolding *|S|* into signed *S*, we revisited tissue-specific gene sets and found that transcript abundance for germline-specific and ubiquitously expressed genes correlated positively with fecundity, whereas transcript abundance for sperm-specific, neuronal, intestinal, hypodermal, somatic, and muscle-enriched genes covaried negatively with fecundity (Fig. 6a). This pattern mirrors the GO enrichment analysis, in which reproductive and cell-cycle-associated processes tended to covary positively with fecundity, while behavioral, neuromuscular, and developmental processes tended to covary negatively (Fig. 5). Together, these results suggest that higher fecundity in the laboratory environment is associated with increased reproductive gene expression and reduced expression of somatic physiological and developmental programs.

The contrast between germline- and sperm-specific expression was especially notable because *C*. *elegans* reproduction depends on a developmental transition from spermatogenesis to oogenesis. In self-fertilizing hermaphrodites, sperm are produced during late larval development before an irreversible switch to oocyte production at adulthood (Ward and Carrel 1979). While models suggest that sperm production limits lifetime fecundity in *C*. *elegans* under optimal conditions (Cutter 2004), experimental evidence indicates that stressful environments may mitigate these constraints (Murray and Cutter 2011). To explore further, we reapplied genotypic selection analysis to test for covariance between fitness and time to maturity—a key determinant of sperm production (Cutter 2004; Murray and Cutter 2011)—predicted by strain-specific transcriptome data (Zhang et al. 2022). Although we detected no significant linear relationship between time to maturity and fecundity (*S* = 0.002955 ± 0.018596, *P* = 0.874; Fig. S9), a signature of weak but significant stabilizing selection was observed (*C* = −0.08034 ± 0.0312, *P* = 0.0108; Fig. 6b). These findings suggest that the CaeNDR population is near a fitness optimum for maturation timing, where tradeoffs between sperm and egg production are modulated by generation time. Increased investment in sperm production likely enhances fitness only up to a critical threshold, beyond which extended generation times incur fecundity fitness costs (Cutter 2004; Murray and Cutter 2011).

To identify the genes mediating these putative tradeoffs in maturation timing, we examined genes with dynamic expression patterns during adolescence (Snoek et al. 2014). Two clusters of genes whose expression displayed the strongest negative covariance with fecundity fitness were identified (Kruskal–Wallis *χ*^2^ = 1078.4, *P* < 2.746 × 10^−4^; Fig. 6c). These clusters were enriched for GO terms associated with molting (e.g. cuticle development, molting cycle, etc.) and the completion of the nematode body plan (e.g. gland morphogenesis, sensory organ morphogenesis, etc.; Figure 6d and e). This pattern reinforces the notion that adolescence redirects energy investment from organismal development toward reproduction.

### TF Activity as a Correlate of Fitness

The preceding analyses suggested that transcript level-fitness covariance is distributed across highly regulated target genes rather than concentrated among TFs with many predicted targets. This raised a related question: even if expression of TF-encoding genes themselves does not show unusually strong covariance with fecundity, could the expression of their downstream regulons reveal TF activities associated with fitness? To address this, we used curated TF-target relationships (Suriyalaksh et al. 2022) and tested whether the target genes for each TF showed significant transcript level-fitness covariance patterns that differed from the GRN target gene background.

**Fig. 7.**
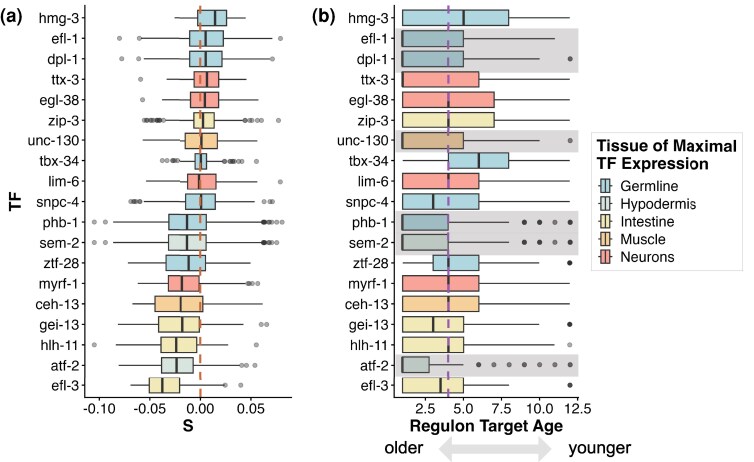
Patterns of transcript expression-fitness covariance highlight the activity of a subset of transcription factors. a) Nineteen transcription factors (TFs) have target genes whose average transcript expression-fitness covariance (*S*) diverges from the null distribution (Mann–Whitney *U*-test, two-sided, *P* < 2.517 × 10^−5^). Boxplots show the distribution of *S*-values (median and interquartile range) among target genes for each candidate TF regulon. Vertical dashed orange line at *S* = 0. b) Average age of each TF's regulon as derived from the phylostrata (PS) values determined by Ma and Zheng (2023) and depicted in Fig. 4. Vertical dashed purple line represents the median phylostratum value for all genes in the genome (PS = 4). Gray shading marks TF regulons significantly enriched for older target genes based on size-matched permutation tests against the GRN target background. Boxes represent the median and interquartile range and are colored by the tissue in which each TF exhibits highest expression as determined by Serizay et al. (2020).

After Bonferroni correction (*P* < 2.517 × 10^−5^), we identified as candidates 19 TFs whose activity might be correlated with variation in fecundity across the CaeNDR population (Fig. 7a). Of these, 10 had annotated mutant or RNA interference phenotypes in WormBase linked to reproductive output (e.g. sterile, reduced brood size, egg-laying defective, fewer germ cells, etc.), underscoring their potential contributions to organismal fitness. For example, expression of the *EGg-Laying defective-38 (egl-38)* regulon was positively associated with fecundity (Fig. 7a). *Egl-38* encodes a paired box (Pax) TF that mediates patterning of the uterine lining and vulva and regulates the expression of key neuropeptides involved in the timing of egg laying (Chamberlin et al. 1997; Webb Chasser et al. 2019). Mutants of *egl-38* are unable to lay eggs and accumulate germline cell corpses, reflecting its essential role in reproduction (Trent et al. 1983; Park et al. 2006; Rajakumar and Chamberlin 2007).

Given that expression of older (Fig. 5) and tissue-specific (Fig. 4) genes covaried positively with fecundity, we reasoned that these TFs might exhibit tissue-specific enrichment and regulate suites of older genes. Consistent with the observation that muscle-enriched genes experienced average levels of covariance with fitness, activity of only two of the 19 TFs was enriched in the musculature (Fig. 7a). By contrast, activity of the highest proportion (8/19) of the TFs was enriched in the germline, followed by activity of those enriched in neurons (4/19) and the intestine (4/19), aligning with these tissues' transcriptomes exhibiting strong signals of fitness covariance when considering both *S* and *|S|* (Figs. 4c and 6a).

Finally, we tested whether candidate TF regulons were enriched for evolutionarily older target genes. Because simple comparison to the genome-wide average can be sensitive to regulon size, we used size-matched permutation tests in which each candidate regulon was compared to random regulons of equal size drawn from the GRN target background. A subset of candidate regulons, including those of *phb-1*, *sem-2*, *efl-1*, *dpl-1*, and *unc-130*, were significantly enriched for older target genes after multiple testing correction (Fig. 7b). However, this pattern was not universal across candidate TFs. Thus, fecundity-associated TF activity appears to include both deeply conserved regulatory programs and more specialized regulons, rather than reflecting a single global bias toward older target genes.

## Discussion

Understanding how patterns of gene expression evolve, and what features of GRNs contribute to their evolutionary dynamics, are central questions in evolutionary biology (Carroll 2008; Romero et al. 2012). However, most changes in gene expression have no major impact on organismal fitness and emerge as a consequence of genetic drift (Lynch 2007). Methodologies that can discriminate between adaptive evolution and genetic drift are essential for uncovering the selective forces shaping GRNs and provide critical insights into the genetic basis of phenotypic diversity (Price et al. 2022; Stinchcombe and Kelly 2025). In this study, we quantified associations between transcript abundance and fecundity of wild-collected *C*. *elegans* strains after a shift to a novel laboratory environment, which highlight expression patterns that could contribute to fitness variation, a prerequisite for adaptive evolution (Lande and Arnold 1983; Hendry and Kinnison 1999).

### Transcripts Associated With Nascent Adaptation to a Novel Laboratory Environment

After implementing multiple testing corrections, expression levels of seven transcripts retained significant covariance with fecundity fitness (*S*; Fig. S1). A notable example was a transcript derived from the nuclear hormone receptor-encoding gene *nhr-114*, whose abundance exhibited the strongest, and most significant, negative covariation with fecundity fitness of all transcripts analyzed (Fig. S1g). *Nhr-114* has recently been associated with a QTL for TLF (Zhang et al. 2022). Its expression is induced during vitamin B12 deficiency and participates in feedback loops that protect germline stem cells from oxidative damage (Gracida and Eckmann 2013; Giese et al. 2020; Qin et al. 2022). It is plausible that variation in *nhr-114* expression might be associated with population-level differences in nutritional requirements, or with altered feeding behaviors that are associated with laboratory-adapted strains. This example underscores the potential for nutrient-sensing and metabolic regulation pathways to make adaptive contributions to fitness, particularly in environments with novel selective pressures.

### Relatively Weak Fitness Covariance for Expression of Most Transcripts

Apart from the seven transcripts identified through our univariate analyses, covariance between gene expression and fitness was generally weak, as reflected in both the linear (*S* and *β*, Fig. 1a and Fig. S4c) and quadratic (*C* and *Ɣ*, Fig. 1b and Fig. S4d) selection differentials and gradients, respectively. Importantly, these patterns persisted even after controlling for potential confounders such as population structure, chromosome-scale selective sweeps, and HDRs (Figs. S2 and S5). This indicates that, on a global scale, our estimates of *S* and *C* for each transcript reflect expression level-fitness relationships more than they do artifacts of genomic context. This is consistent with recent observations that genome-wide patterns of allele-specific gene expression are robust to genes' overlap with HDRs in *C*. *elegans* (Bell et al. 2023, 2025).

These findings align with theoretical models of selection on complex traits, which predict that most phenotypic variation is subject to weak selective pressures due to polygenic trait architectures and pleiotropy (Hoekstra et al. 2001; Kingsolver et al. 2001). It is also consistent with observations at microevolutionary timescales in other systems, including ones of weak directional selection on transcript abundance in rice and morning glory plants, salmonid fish, and the red flour beetle *Tribolium castaneum* (Groen et al. 2020; Koch and Guillaume 2020; Ahmad et al. 2021; Gupta et al. 2025; Henry and Stinchcombe 2025), and of trends toward weak stabilizing selection (i.e. *C* < 0) on transcript expression levels in rice (Groen et al. 2020; Gupta et al. 2025). Together, these suggest that weak covariance between fitness and expression levels of most genes is a common feature across species and may reflect the inherent, scale-free properties of GRNs that stabilize transcriptomes through regulatory complexity and buffering mechanisms (Whitacre and Bender 2010; Macneil and Walhout 2011; Whitacre 2012).

### Tissue-specific Patterns of Transcript Level-fitness Covariance

Despite numerous studies examining tissue specificity from a macroevolutionary perspective, whether tissue-specific genes might evolve at different rates on microevolutionary timescales has remained unclear. Using *τ* as a metric of tissue-specific gene expression, we found that the expression of tissue-specific genes covaries more strongly with fecundity fitness of *C*. *elegans* in the laboratory environment (*|S|*; Fig. 4a), suggesting that these genes have the potential to evolve faster at the expression level. This observation agrees with positive correlations between *|S|* and *τ* in rice (Groen et al. 2020).

The magnitude and direction of gene expression-fecundity fitness covariance (*|S|*) also varied across tissues. Neuronal genes showed unexpectedly strong covariance with fecundity (Fig. 3c), possibly reflecting the unique selective pressures laboratory environments impose on the nervous system (Sterken et al. 2015). Intestinal genes also showed relatively strong covariance, paralleling findings in vertebrates, where detoxification-associated organs, such as the kidneys and liver, rapidly accumulate gene expression variation across species (Brawand et al. 2011; El Taher et al. 2021; Mantica et al. 2024). In contrast, germline-enriched genes showed weaker absolute covariance with fecundity fitness (*|S|*; Fig. 3c), but were notable because they exhibited a positive covariance on average (*S* > 0; Fig. 6a). This pattern aligns with the rapid divergence of reproductive transcriptomes reported across diverse taxa (True and Haag 2001; Brawand et al. 2011; Fukushima and Pollock 2020; El Taher et al. 2021; Mantica et al. 2024), while also suggesting that reproductive gene expression may contribute to fecundity in the laboratory environment in a unique way relative to other tissues.

This interpretation is further supported by positive fitness covariance for expression of genes involved in meiosis and oogenesis (Fig. 5). Strains with higher fecundity in the laboratory environment may therefore exhibit increased expression of reproductive programs linked to egg production or reproductive maturation. Similar patterns have been observed in the red flour beetle *Tribolium castaneum*, where strong positive directional selection was detected on expression of *Vitellogenin* genes that produce precursors to egg yolk proteins (Koch and Guillaume 2020). In our analysis, the regulon of *egl-38*, which encodes a Pax TF critical for uterine and vulval development and the timing of egg-laying (Chamberlin et al. 1997; Webb Chasser et al. 2019), also showed a positive covariance between its expression and fitness in the laboratory environment (Fig. 7a). Together, these results suggest that fecundity-associated gene expression variation is concentrated in tissue-specific programs linked to behavior, metabolism, and reproduction.

### Network Architecture and Genome-wide Regulatory Variation

Network position is expected to influence the evolution of gene expression because highly connected genes participate in many regulatory relationships and variation in their expression may have broader downstream consequences (Liao and Zhang 2006; Josephs et al. 2017). Consistent with this expectation, we found that expression of high in-degree genes showed stronger linear covariance with fecundity (Fig. 2e). This pattern suggests that variation in fecundity of *C*. *elegans* in this novel laboratory environment might reflect variation in broader regulatory network state, where highly connected genes act as hubs that integrate upstream genetic, developmental, and physiological variation.

These interpretations are complemented by comparative studies showing how network position influences evolutionary dynamics. In populations of the plant species *Capsella grandiflora* and *Populus tremula*, for example, genes at network cores tend to show stronger constraints, while more peripheral genes accumulate larger regulatory divergence (Josephs et al. 2017; Mähler et al. 2017). Altogether, these observations support a broader view that GRNs filter the relationship between molecular variation and organismal fitness by distributing effects across many interconnected genes (Boyle et al. 2017; Liu et al. 2019).

### TF Activity and Coordinated Covariance With Fitness

We identified 19 TFs whose target genes' expression levels showed strong fitness covariance (Fig. 7a). Many of these TFs have been shown empirically to determine fecundity: consistent with expression patterns of tissue-specific genes (Fig. 4), we found that activity of these TFs tends to be enriched in tissues for which we found stronger fitness covariance, such as the germline, neurons, and intestine (Fig. 7b). Some candidate regulons were also enriched for older target genes (Fig. 7b), suggesting that older genes might covary more with fitness in a novel environment due to their integration into biologically relevant network modules. Together, these findings suggest that activity of a subset of key TFs shapes the regulatory landscape of tissues critical for fitness.

Interestingly, even TFs without defined links to fecundity emerged as promising candidates for functional exploration. For example, we observed a positive expression-fitness covariance for the regulon of the bZIP TF encoded by *zip-3*. Along with its paralog *atfs-1*, *zip-3* encodes a key regulator of the mitochondrial unfolded protein response (mUPR; Fiorese et al. 2016; Deng et al. 2019). Unlike ATFS-1, ZIP-3 functions as a negative regulator, preventing hyperactivation of the mUPR (Deng et al. 2019). Given that mitochondrial dysfunction is a conserved hallmark of aging (Brys et al. 2010; López-Otín et al. 2013) and is tightly linked to aging-related transcriptional changes in *C*. *elegans* (Roux et al. 2023), variation in ZIP-3 activity may play a role in mediating population-level differences in fecundity by balancing stress responses tied to mitochondrial energy status. Future studies investigating the activity of TFs such as ZIP-3 across strains could reveal new insights into the regulation of fecundity in *C*. *elegans*.

## Conclusion

Our findings highlight how patterns of gene expression-fitness covariance in *C*. *elegans* upon transition to a novel (laboratory) environment are shaped by GRN architecture, tissue specificity, and regulatory variation. While network constraints limit variance in the expression of core genes, regulatory variants and chromatin dynamics provide opportunities for transcriptome shifts in response to novel environments. Although our estimates do not represent direct measurements of natural selection, they reveal how the same principles governing GRN evolution over macroevolutionary timescales can also structure gene expression in contemporary populations. In this way, following adaptive processes in artificial environments such as the laboratory can serve as a tractable context for dissecting how genomic and regulatory mechanisms channel phenotypic diversity.

## Methods

### Univariate Genotypic Selection Analyses

To assess patterns of fitness covariance for expression levels of genes across the *C. elegans* genome, we estimated total (direct and indirect) linear (*S*) and quadratic (*C*) selection differentials for the abundance of 25,849 transcripts (representing approximately 16,094 genes) in a population of 207 genetically diverse wild-collected strains of the *Caenorhabditis elegans* Natural Diversity Resource (CaeNDR)—as quantified by (Zhang et al. 2022) from synchronized, laboratory-reared worms of each strain at the young adult stage—using the *glm()* function in R (Rausher 1992). To preprocess the genome-wide gene expression data for analysis, we excluded expression information from 14 strains for which no TLF data were available. As a fitness component metric, we normalized TLF (*normTLF*) from worms reared in the same laboratory environment as reported by (Zhang et al. 2021). We did this by dividing TLF of each strain by the population mean. After this, we standardized the abundance (in normalized, log-transformed transcripts per million, log(TPM + 0.5)) of each transcript (*stand_expr*) by subtracting the population mean and dividing by the population standard deviation for that transcript. For linear selection (*S*) models, we provided *glm()* with the formula `*normTLF ∼ stand_expr`*, and for quadratic selection *(C)*, we provided the formula `*normTLF ∼ stand_expr + I(stand_expr^2^)`*. Estimates of *C* were then multiplied by 2 (Rausher 1992). Bonferroni correction was performed to correct for multiple testing using the *p*.*adjust()* function in R.

To assess the impact of population structure on the genotypic selection models, we retrieved information on the location of origin of each strain from the CaeNDR database (https://caendr.org). We re-estimated selection differentials after identifying, and excluding from the analysis, 14 Hawaiian strains that are genetically divergent (Crombie et al. 2019) and collectively exhibit lower fecundity under laboratory conditions (Zhang et al. 2021). We further re-estimated selection differentials using only the European strains in the database.

We additionally estimated total selection differentials for time to maturity, as predicted from the strain-specific transcriptomes reported by (Zhang et al. 2022) following the same procedures.

### Multivariate Genotypic Selection Analyses

We estimated direct linear (*β*) and quadratic (*Ɣ*) selection gradients on coordinated patterns of gene expression across *C*. *elegans* strains by performing PC analysis using the *prcomp()* function in R. We selected the top 22 PCs that individually explained at least 0.5% of the population variance in genome-wide gene expression and retrieved loading values for the abundance of each transcript per strain into PC1-PC22. We subjected the strain-specific loading values to multivariate analysis using the *glm()* function in R with the formulas *`normTLF ∼ PC1*  *+*  *PC2*  *+*  *…*  *+*  *PC22` (β)* and *`normTLF ∼ PC1*  *+*  *I(PC1^2^)*  *+*  *PC2*  *+*  *I(PC2^2^)*  *+*  *…*  *+*  *PC22*  *+*  *I(PC22^2^)` (Ɣ)*. Estimates of *Ɣ* were then multiplied by 2 (Rausher 1992).

To obtain biological insight from PCs of interest, we retrieved the loading values for the abundance of each transcript into PC1-PC22. We considered the top 200 transcripts per PC for further analysis.

### GO Analyses

To determine whether groups of transcripts related to particular functional categories stood out for patterns in expression-fitness covariance, we retrieved GO annotations for each gene from the Ensembl WormBase ParaSite BioMart release 19 (https://parasite.wormbase.org/biomart/martview) *C*. *elegans* (PRJNA13758, WS290) dataset. We merged our genotypic selection models with GO annotations and determined functional categories with distinct average transcript abundance-fitness covariance levels compared to the total genome-wide gene expression dataset using two-sided Mann–Whitney *U*-tests with the *wilcox*.*test()* function and adjusting the *P*-values using Bonferroni correction with the *p*.*adjust()* function in R.

Further gene set enrichment analyses (GSEAs) on smaller subsets of genes were performed using the ShinyGO v0.81 web server with all transcripts showing expression across the strains supplied as background (Ge et al. 2020).

### The Influence of Network Architecture on Transcript Expression-fitness Covariance

To determine how network position impacts the strength and pattern of transcript expression-fitness covariance, we retrieved information on ACRs collected from bulk larval stage 4 (L4) worms in addition to ACRs from flow-sorted epidermal, hypodermal, intestinal, muscle, and germline nuclei (Serizay et al. 2020). ACRs, defined for 20,222 genes, were classified as promoters or enhancers based on genomic location (Serizay et al. 2020).

We also retrieved gene regulatory information from a consensus GRN built off of time-course RNA-sequencing data from aging worms (Suriyalaksh et al. 2022). The GRN included 313,562 interactions between 590 TFs and 7,564 target genes. To enhance inference of TF-target relationships, the GRN was informed with gold-standard chromatin immunopurification-sequencing, yeast one hybrid assays, TF binding site analyses, and TF knockdown gene expression analyses (Suriyalaksh et al. 2022). We inferred in-degree as the number of TFs regulating a target and out-degree as the number of targets regulated by each TF.

To account for both the number of ACRs and GRN properties, we grouped genes into functional categories, ensuring that the size of each group was similar so that differences between the classes were minimized. We compared average selection differentials across the various regulatory categories using Kruskal–Wallis tests (*kruskal_test())* followed by Dunn's post hoc tests (*dunnTest()*) in R.

To estimate TF activity-fitness covariance, we determined groups of TF targets with significantly distinct average *S*-values relative to the set of 7,564 target genes using two-sided Mann–Whitney *U*-tests with the *wilcox*.*test()* function and adjusting the *P*-values using Bonferroni correction with the *p*.*adjust()* function in R. We determined the tissue of maximal expression using RNA-sequencing data from flow-sorted epidermal, hypodermal, intestinal, muscle, and germline nuclei described by (Serizay et al. 2020).

### The Influence of Tissue Specificity on Transcript Expression-fitness Covariance

To determine how tissue specificity impacted the strength of transcript expression-fitness covariance, we reanalyzed published transcript profiles for 20,222 genes across flow-sorted epidermal, hypodermal, intestinal, muscle, and germline nuclei (Serizay et al. 2020). For each gene, we determined the tissue specificity index *τ* (tau):


$$\tau \;=\frac{\sum_{i=1}^{n}\;(1-x_{i})}{n\;}$$

where *n* is the number of tissues profiled and *x_i_* is the normalized expression of a gene in a given tissue (Yanai et al. 2005). We grouped genes into 10 equally sized bins of tissue specificity and compared *S*-values across these bins using Kruskal–Wallis tests (*kruskal_test())* followed by Dunn's post hoc tests (*dunnTest()*) in R.

We retrieved information on genes expressed specifically in each profiled tissue: epidermis, hypodermis, intestine, muscle, germline, and sperm (Serizay et al. 2020). We then determined 95% confidence intervals for the distributions of *S* and *|S|* for tissue-specific gene sets and identified tissues facing stronger- or weaker-than-average selection compared with the genome-wide confidence intervals.

### The Influence of Gene Age on Transcript Expression-fitness Covariance

We evaluated the role of gene age in shaping the strength and pattern of transcript expression-fitness covariance by employing a phylostratigraphy approach. In phylostratigraphy, proteomes of various species are aligned and underlying genes are partitioned into phylostrata (PS) dependent on the presence of homologs within particular clades. We downloaded PS values for each gene in the *C*. *elegans* genome from a recent study (Ma and Zheng 2023) and compared *S* across each PS using Kruskal–Wallis tests (*kruskal_test())* followed by Dunn's post hoc tests (*dunnTest()*) in R.

### Linking Historical Selection to Fitness Covariance in a Novel Environment

We compared our estimates of transcript expression-fitness covariance—as a proxy for the microevolutionary response of transcript abundance to selection in a novel laboratory environment (Lande and Arnold 1983; Hendry and Kinnison 1999)—with rates of genome and protein-coding sequence evolution to analyze if historical selection may influence how variation in gene expression can continue to impact organismal fitness. Specifically, we retrieved *dN/dS* values from (Ma et al. 2024), who calculated protein sequence divergence for *C*. *elegans* relative to 11 congeneric species. We also recalculated Tajima's *D* values for the *C*. *elegans* population using VCF files for each strain obtained from (Lee et al. 2021), in 1-kb bins across the genome. We retrieved genomic locations for genes from the Ensembl WormBase ParaSite BioMart release 19 (https://parasite.wormbase.org/biomart/martview) *C*. *elegans* (PRJNA13758, WS290) dataset and assigned Tajima's *D* values to genes based on the average Tajima's *D* values across each gene's body, promoter, and enhancers.

We compared *dN/dS* and Tajima's *D* values across genes of differing ages, tissue specificities, and network properties using the aforementioned statistical tests in R.

## Supplementary Material

evag180_Supplementary_Data

## Data Availability

Normalized genome-wide gene expression and TLF values across 207 *C*. *elegans* strains were retrieved from Data File S1 from (Zhang et al. 2022). Expression QTL data were retrieved from Data File S2 from (Zhang et al. 2022). Chromatin accessibility information, tissue-specific gene expression values (in TPM), and tissue-specific gene sets were retrieved from Table S2 from (Serizay et al. 2020). For our GRN, we used the “max AUFE” model from Table S6A from (Suriyalaksh et al. 2022) after filtering out non-TF regulators, including the insulin receptor DAF*-2*, and chromatin cofactors. Phylostratigraphy values were retrieved from Table S1 from (Ma and Zheng 2023). *dN/dS* values for *C*. *elegans* were retrieved from Table S4 from (Ma et al. 2024). Tajima's *D* values and genomic coordinates of HDRs in *C*. *elegans* as calculated by (Lee et al. 2021) were downloaded from GitHub at https://github.com/AndersenLab/Ce-328pop-div. Scripts to repeat the analyses, and all computations reported here, are available on GitHub at https://github.com/tylerinskeep/expression-fitness-covariance-celegans, and in the Supplementary Materials.
