# Supplementary material for: Network Properties Influence Covariance Between Gene Expression and Fecundity Fitness of Caenorhabditis elegans in a Novel Laboratory Environment
Source: Genome Biol Evol. 2026 Aug 3;18(8):evag180. doi: 10.1093/gbe/evag180 (PMC13429268; doi:10.1093/gbe/evag180)

**Supplementary Figure S1. Linear relationships between transcript abundance and fecundity fitness are largely correlated across standard laboratory culture conditions. A.** Correlation of total lifetime fecundity was moderate across measured laboratory environments (Spearman *⍴ = 0.352, p = 0.003715*)*.* **B.** Linear gene expression-fecundity covariances are largely consistent when considering fitness in both environments (Spearman *⍴ = 0.632, p < 2.2x10^-16^*)*.* **C.** Nonlinear gene expression-fecundity covariances, however, are not (Spearman *⍴ = 0.299, p < 2.2x10^-16^*).

**Supplementary Figure S2. Transcripts with abundances that show significant fitness covariance in the laboratory environment.** Linear regressions of fecundity fitness on abundances of seven transcripts that retained significant linear selection differentials (*S*) following Bonferroni correction (*p < 3.87 x 10^-7^*), including transcripts encoding **A.** a UDP-glucuronosyltransferase involved in detoxification of dietary metabolites (*ugt-9)*, **B.** a predicted Zinc ion-binding protein (*B0348.2*), **C.** a major component of the muscle actin filament (*Titin, ttn-1*), **D.** the *C. elegans* homolog of the human Fe65 protein implicated in Alzheimer’s disease (*feh-1*), **E.** an uncharacterized C-type lectin (*clec-37*), **F.** an uncharacterized protein with a predicted role in lipid metabolism (*F25A2.1*), and **G.** a nuclear hormone receptor that buffers germline stem cells from oxidative damage (*nhr-114*).

**Supplementary Figure S3. Genotypic selection models capture fecundity-associated quantitative trait loci. A-D.** Volcano plots showing transcript-level associations between gene expression and total lifetime fecundity in standard laboratory growth conditions. Columns show results for the linear transcript expression-fitness covariance term (*S*, **A, C**) and the population expression variance-fitness covariance term (*C*, **B, D).** The top row shows models fit using fecundity measured in liquid culture, while the bottom row shows models fit using fecundity measured on agar plates. Orange points indicate transcripts encoded by genes located within quantitative trait loci (QTL) for total lifetime fecundity previously identified by Zhang et al. (2022), while gray points indicate all other transcripts. Horizontal dashed lines mark the nominal significance threshold for each model. In the liquid-culture analysis, ten fecundity QTL encoded at least one transcript with significant *S*, and two QTL encoded at least one transcript with significant *C.*

**Supplementary Figure S4. Principal component-based genotypic selection models identify fecundity-associated axes of transcriptome variation. A, B.** Principal component (PC) analysis was used to summarize correlated transcript abundance patterns across *C. elegans* strains. **A.** Cumulative variance explained by PCs. **B.** Proportion of variance explained by each PC. PC1-PC22 were retained for multivariate genotypic selection analysis. **C, D.** Estimated selection gradients for retained PCs. **C.** Linear selection gradients (*β*) quantify the association between each transcriptomic axis and total lifetime fecundity in standard laboratory culture conditions. **D.** Quadratic selection gradients (*ɣ*) quantify the association between variation along each transcriptomic axis and total lifetime fecundity. Bars show estimated gradients with standard error bars; colors indicate -log10 adjusted *p*-values. Significant PCs were selected for downstream functional interpretation. **E-K.** Gene loadings were ranked for each significant PC, and the top 200 genes with the strongest loadings were used for Gene Ontology (GO) enrichment analysis. PC8 and PC11 did not show significant enrichment for any ontologies among the top-loading genes.

**Supplementary Figure S5. Population structure and genomic features have limited effects on transcript level-fitness covariance estimates. A, B.** Univariate genotypic selection models were recalculated using reduced *C. elegans* strain sets to assess sensitivity to population structure. **A.** Models excluding genetically divergent Hawaiian strains retained 176 non-Hawaiian strains. **B.** Models using only European strains retained 99 strains. Maps show the geographic distribution of strains included in each analysis. Scatterplots compare transcript level-fitness covariance estimates (*S*) from each analysis to estimates from the global strain panel. Estimates were highly concordant with the global analysis in both instances (Spearman *𝜌 > 0.8075, p < 2.2x10^-16^*). **C.** Genomic distribution of the strength of fitness covariance (*|S|*). A linear model testing chromosome, chromosomal domain, and their interaction found significant variation in *|S|* among chromosomes, and a weaker but significant effect of chromosomal domain, but no chromosome-by-domain interaction. **D.** To determine whether selective sweep history drives transcript level-fitness covariance patterns, each gene was characterized by its sweep burden, defined as the number of strains in which that gene overlapped a swept haplotype based on Lee et al. (2021). Genes were then grouped into five equal-sized bins by sweep burden. *|S|* did not differ across genes with variable sweep burden (linear model, *|S| ~ sweep_burden*, *p = 0.0861*), suggesting that selective sweeps do not explain the observed genome-wide patterns of transcript level-fitness covariance. **E.** To test whether overlap with hyperdivergent regions (HDRs) biases transcript level-fitness covariance estimates, segregating HDRs were identified across the 172-strain panel, and each gene was assigned an HDR burden, defined as the number of strains in which that gene overlapped an HDR. Because transcript abundance estimates for genes within strain-specific HDRs can be unreliable and must be excluded from those strains (Lee et al. 2021; Zhang et al. 2022), estimates of *|S|* for genes overlapping common HDRs are estimated from smaller subsets of the population. Genes overlapping HDRs were categorized based on the HDR’s frequency. *|S|* did not differ significantly among HDR burden categories (linear model, *|S| ~ HDR_category, p = 0.341*), suggesting that HDRs, and the uncertainty of gene expression estimates that accompany them, do not explain genome-wide patterns of transcript level-fitness covariance.

**Supplementary Figure S6. Regulatory architecture is differentially associated with transcript level-fitness covariance, protein evolution, and nucleotide variation.** Genes were grouped by features of regulatory architecture, including promoter and enhancer chromatin accessibility, gene regulatory network (GRN) in-degree, and transcription factor (TF) out-degree. Across each regulatory feature, we compared the magnitude of transcript level-fitness covariance (*|S|*), coding sequence evolutionary rate (*dN/dS*), and within-species nucleotide variation (Tajima’s *D*). **A.** Genes with increasingly accessible promoter chromatin did not deviate from genome-wide patterns of nucleotide diversity, as measured by Tajima’s *D* (*p = 0.9392*). **B-D.** Genes with increasingly accessible enhancer chromatin showed **B.** stronger transcript level-fitness covariance (*|S|*, *p < 2.2x10^16^*), and **C.** lower coding sequence evolutionary rates (*dN/dS*; *p < 2.2x10^-16^*), but **D.** did not deviate from genome-wide patterns of nucleotide diversity (Tajima’s *D*, *p = 0.1859*). **E-H.** GRN position was also compared against the same parameters. **E.** Genes regulated by more TFs did not deviate from genome-wide patterns of nucleotide diversity (Tajima’s *D,* Mann-Whitney *U-*test, *p = 0.2324*). **F-H.** TFs with higher out-degree, defined by the number of target genes one regulates, did not differ in **F.** transcript level-fitness covariance (*|S|*; *p = 0.3756*), **G.** coding sequence evolutionary rate (*dN/dS; p = 0.4346*), or **H.** nucleotide diversity (Tajima’s *D*; *p = 0.2331*). Boxplots show the median and interquartile range. For comparisons with more than two groups, overall differences were tested using Kruskal-Wallis tests, followed by Dunn’s post hoc tests with Bonferroni correction. Letters indicate post hoc significance groups; groups that do not share a letter differ at adjusted *p < 0.05*, with *a* assigned to the group with the lowest median. For two-group comparisons, significance was assessed using Mann-Whitney *U* tests.

**Supplementary Figure S7. Tissue-specific genes evolve faster at the sequence level. A.** Distribution of *τ* (tau) values as determined from tissue-specific transcriptome data of L4-stage worms (Serizay et al. 2020). Most transcripts exhibit tissue-enriched expression patterns. **B.** Genomic regions encoding tissue-enriched transcripts do not experience large deviations from genome-wide patterns of purifying selection (𝜒^2^ = 20.883, *p = 0.01318*). **C.** Genes with tissue-specific expression patterns evolve faster at the coding sequence level than those with constitutive expression patterns (𝜒^2^ = 248.74, *p < 2.2x10^-16^*). Boxplots show the median and interquartile range. For comparisons with more than two groups, overall differences were tested using Kruskal-Wallis tests, followed by Dunn’s post hoc tests with Bonferroni correction. Letters indicate post hoc significance groups; groups that do not share a letter differ at adjusted *p < 0.05*, with *a* assigned to the group with the lowest median.

**Supplementary Figure S8. Younger genes are more likely to show tissue-specific expression and have less complex regulatory grammar. A.** Younger genes are more likely to adopt tissue-specific expression profiles (Kruskal-Wallis 𝜒^2^ = 1352.8, *p < 2.2x10^-16^*). **B.** Younger genes have fewer accessible chromatin regions (ACRs) in their promoters (Kruskal-Wallis

𝜒^2^ = 650.69, *p < 2.2x10^-16^*). Boxplots show the median and interquartile range. For comparisons with more than two groups, overall differences were tested using Kruskal-Wallis tests, followed by Dunn’s post hoc tests with Bonferroni correction. Letters indicate post hoc significance groups; groups that do not share a letter differ at adjusted *p < 0.05*, with *a* assigned to the group with the lowest median.

**Supplementary Figure S9. No linear covariation between maturation timing and fecundity fitness.** Maturation timing does not show evidence of experiencing directional selection, as suggested by the lack of a significant association with total lifetime fecundity (*S = 0.002955 ± 0.018596, p = 0.874*).


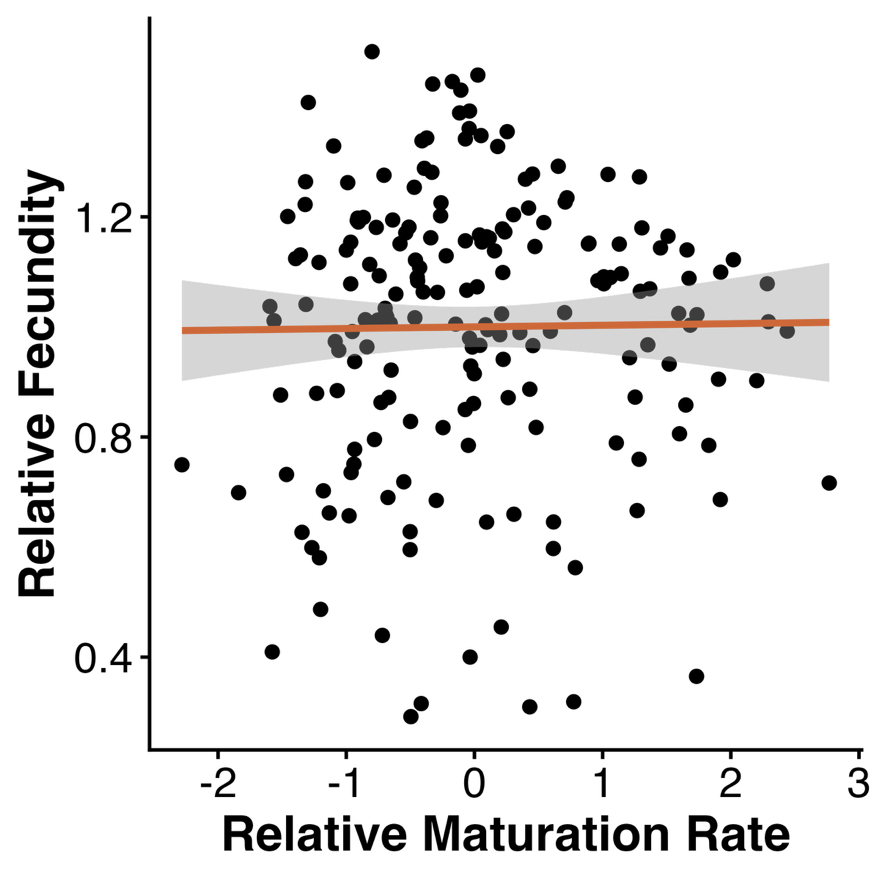

Supplement: evag180_Supplementary_Data [file evag180_supplementary_data.zip › SupplementaryFigures.docx]
